# PHENOME-WIDE SEARCH OF COGNITIVE RESERVE PROXIES ACROSS AGE COHORTS

**DOI:** 10.1093/geroni/igac059.2952

**Published:** 2022-12-20

**Authors:** Anqing Zheng, Sally Wadsworth, Naomi Friedman, Chandra Reynolds

**Affiliations:** University of California, Riverside, riverside, California, United States; University of Colorado Boulder, Boulder, Colorado, United States; University of Colorado, Boulder, Boulder, Colorado, United States; University of California, Riverside, Riverside, California, United States

## Abstract

Cognitive reserve (CR) refers to adaptability allowing for better cognitive outcomes given the degree of brain changes or other risk factors for cognitive decline. Despite significant research efforts, our knowledge of cognitive reserve proxies remains limited. Studies predominantly use a single sociodemographic variable (e.g., education attainment) as a proxy measure when CR can manifest in multiple domains. Studies also tend to rely on older samples, whereas adversity factors of cognitive performance may have differing onset ages, suggesting different risk or protective mechanisms at different ages. We examine two cohort datasets spanning from early adolescence (Adolescent Brain Cognitive Development study, N = 5559) up to the cusp of mid-adulthood (Colorado Adoption/Twin Study of Lifespan behavioral development and cognitive aging; CATSLife, N = 1327) to evaluate the role of CR proxies across over 100 variables. Defined as a moderator between a cognitive outcome and structural brain measure, these variables cover behavioral, environmental, physical and mental health, and psychological trait domains. Using cross-validated regularized regression, we identified dozens of factors acting as CR proxies (e.g., leisure reading as protective and screen usage as a risk factor during adolescence). We then use a within-family design to examine the moderation effect over and above genetic and environmental covariates. For example, adolescents who read more tend to show better cognitive performance given their gray matter volume (b = .093, 95%CI = [0.035, 0.152]). This study aims to identify factors that could be targeted with scalable prevention and intervention efforts earlier in life to maximize cognitive functioning.

